# Capitolunate arthrodesis versus four-corner fusion for advanced wrist collapse: a systematic review and meta-analysis

**DOI:** 10.1186/s13018-026-06747-x

**Published:** 2026-03-13

**Authors:** Mohammad Al-Badaineh, Rachel X. Shi, James L. Cross, Om B. Jahagirdar, John Slevin, Julie Mekhail, Xuan Luo, Motasem H. Salameh

**Affiliations:** 1 Faculty of Medicine, Jordan University of Science and Technology, Irbid, 22110 Jordan; 2 Yale School of Medicine, 333 Cedar St, New Haven, CT 06510 USA; 3 Frank H. Netter MD School of Medicine, Quinnipiac University, North Haven, CT USA; 4 Department of Orthopaedics and Rehabilitation, Yale School of Medicine, New Haven, CT USA; 5 Chicago Medical School , Rosalind Franklin University , Chicago, IL USA

## Abstract

**Purpose:**

Capitolunate fusion (CLF) has been proposed as a viable alternative to other motion-preserving techniques such as three-corner fusion (3CF) and four-corner fusion (4CF) for scapholunate advanced collapse (SLAC) and scaphoid nonunion advanced collapse (SNAC). This study systematically reviewed the literature comparing clinical outcomes and complication profiles of CLF versus 4CF; a small number of three-corner fusion cases were included.

**Methods:**

The PubMed, EMBASE, and Web of Science databases were systematically searched for articles published between 1990 and 2025. Two independent authors performed blinded screening of titles and abstracts, followed by blinded full-text review. Outcomes of interest included the visual analog scale (VAS) score, Patient-Rated Wrist Evaluation (PRWE), grip strength, Disabilities of the Arm, Shoulder and Hand (DASH) score, reoperation rate, adverse events, and range of motion.

**Results:**

Seven studies were included, consisting of six retrospective cohort studies and one randomized controlled trial, encompassing a total of 320 patients. The VAS score demonstrated a borderline non-significant standardized mean difference of 0.34 (95% confidence interval: − 0.004 to 0.69; *p* = 0.053) in favor of four corner fusion. No statistically significant differences were observed between CLF and 4CF for PRWE, DASH score, range of motion, overall complication rate, reoperation rate, or nonunion rate. Only seven patients underwent three-corner fusion, precluding subgroup-specific analysis.

**Conclusions:**

Compared with four-corner fusion, capitolunate fusion yields comparable patient-rated outcome measures, strength, motion, and complication profiles. Higher-quality prospective studies are required to further validate these findings.

**Level of evidence IV:**

Therapeutic study.

**Supplementary Information:**

The online version contains supplementary material available at 10.1186/s13018-026-06747-x.

## Introduction

Scapholunate advanced collapse (SLAC) and scaphoid nonunion advanced collapse (SNAC) can lead to considerable pain and disability owing to the development of radiocarpal and midcarpal arthrosis [1–4]. Currently, there are no established surgical procedures capable of reconstructing damaged cartilages. Salvage procedures can be employed to reduce pain while maintaining some motion [5]. These procedures include partial wrist fusion techniques, such as three-corner fusion (3CF), four-corner fusion (4CF), proximal row carpectomy (PRC), including variants with radiocarpal prosthetic interposition (PRC + RCPI), and capitolunate fusion (CLF). Of these options, 4CF is one of the most widely used techniques for the treatment of SLAC or SNAC [6]. 4CF has been demonstrated to improve pain relief and grip strength for satisfactory long-term functional outcomes but is linked to decreased range of motion (ROM) and a high rate of nonunion [7, 8]. Recent literature suggests that 3CF and CLF may outperform 4CF by providing improved postoperative wrist arc of motion and patient-reported outcome measures while maintaining similar union rates and complications [9–12].

Restricting fusion of the lunate and capitate is theoretically less invasive than 4CF and PRC and allows for a simpler, more time-efficient surgical procedure that maintains adaptive triquetral motion during ulnar and radial deviation of the wrist [13]. In the largest prior systematic review by Dunn et al. of 80 patients across 6 studies showed that CLF is a viable option for SLAC and SNAC wrists, with complications similar to those seen in 4CF [6]. Historically, CLF demonstrated concerns of non-union, possibly due to inadequate immobilization time and the use of silicone scaphoid-replacing implants, as demonstrated in a cohort study of 18 patients by Kirshenbaum et al. from 1993 [14]. However, recent evidence by Elshahhat et al. suggests contrary findings, with similar complication rates between two cohorts of 31 CLF and 34 4CF patients [15].

Although the most recent literature demonstrates that CLF achieves comparable outcomes with other operations for SLAC and SNAC, the largest systematic review and cohort studies to-date contain only 80 and 65 patients, respectively [6, 15]. Therefore, it is difficult to formulate a powerful consensus on optimal surgical treatment. In the largest sample to-date of 320 patients across 7 studies, this systematic review/meta-analysis aimed to review recent literature evaluating the efficacy of CLF against other techniques and examine indicators, outcomes, and potential complications. This study aims to provide the most comprehensive evidence available to aid hand surgeons in the decision-making process by contextualizing the current literature.

## Methods

This study adheres rigorously to the Preferred Reporting Items for Systematic Reviews and Meta-Analyses (PRISMA) guidelines [16] and is registered with the PROSPERO International Prospective Systematic Review Database (registration number: CRD42025640131).

### Selection criteria

The articles included in this study examined the primary intervention of capitolunate fusion (CLF) in comparison with alternative interventions such as four-corner fusion (4CF) or three-corner fusion (3CF). The eligible study types included randomized controlled trials, prospective cohort studies, retrospective cohort studies, and case-control studies. The primary outcomes were range of motion (ROM), radiographic union rate, and visual analog scale (VAS) pain. The secondary outcomes were the patient-rated wrist evaluation (PRWE) score, disabilities of the arm, shoulder, and hand (DASH) score, grip strength, reoperation rates, and complication rates. Included studies had to report on at least one primary outcome measure. Other inclusion criteria were an adult patient population (≥ 18 years old) with at least 10 patients assessed, and a minimum follow-up period of six months, chosen to permit evaluation of early clinical and radiographic outcomes while avoiding exclusion of smaller comparative series.

### Data sources and searches

We performed a systematic search on December 31, 2024, of the PubMed, EMBASE, and Web of Science databases for articles published from January 1990 onward using the following search term (syntax modified depending on database): ((“Scaphoid nonunion advanced collapse” OR “Scapholunate advanced collapse” OR “Scaphoid Bone” OR “Osteoarthritis*”) AND (“CLA” OR “Capitolunate arthrodesis” OR “Capitolunate Arthrodeses” OR “Lunocapitate Arthrodesis” OR “Lunocapitate Arthrodeses” OR “fusion*”)) AND (“Pain-free” OR “Pain free” OR “Pain score” OR “Complicat*” OR “Musculoskeletal Pains” OR “Musculoskeletal Pain”).

### Study selection

Two authors (M.A and R.S) performed a blinded initial screening of article abstracts identified through the search. The manuscripts then underwent blinded full-text screening in accordance with previously outlined inclusion criteria. Disagreements were resolved by a third author (M.S).

### Data extraction and analysis

Two authors (M.A and R.S) independently extracted all the data from the selected studies, including study details, demographic data, intervention measures, and outcome measures. A plot digitizer tool was used to extract data from studies that exclusively reported data in figures (e.g., box-and-whisker plots). All reported medians and interquartile ranges (IQRs) were approximated as means and standard deviations, under the assumption that the overall distributions did not significantly deviate from a normal distribution. Discrepancies were resolved by consensus involving two additional independent reviewers (J.S. and J.M.).

The data were synthesized for pooled analysis considering clinical and methodological variations between studies and statistical heterogeneity. The outcome measures of the visual analog scale (VAS) score, Patient-Rated Wrist Evaluation (PRWE), grip strength, Disabilities of the Arm, Shoulder and Hand (DASH) score, reoperation rate, adverse events and complications (i.e., nonunion rate), and range of motion (flexion, extension, ulnar deviation, radial deviation, pronation, and supination) were included in our systematic review.

For each study, demographic differences between CLF and 3CF/4CF were assessed using Welch’s t-test for patient age and chi-squared tests for sex, procedure laterality, smoking history where sufficient data were available.

Measures for which pre- and post-intervention data were available for more than three studies were grip strength, VAS score, patient-rated wrist evaluation, DASH score, and ROM. For each intervention, the pre- and post-intervention mean differences and pooled standard deviations were computed for each study, where sufficient data were available. A sensitivity analysis was performed for three different estimates of the within-subject correlation coefficient (*r* = 0.3, 0.5, and 0.7).

For each measure of every study, the Hedge’s g effect size and standard error of 3CF/4CF relative to CLF on the pre-/post-intervention mean difference were calculated. Furthermore, utilizing the pre- or post-intervention mean difference and pooled standard deviation for each study, a random effects model was used to calculate the overall effect and heterogeneity metrics (Q and I^2^) for each measure across studies [17]. Statistical significance was defined as *p* < 0.05.

### Study quality and risk of bias

The quality of each study was independently assessed by two authors (M.A and R.S) using the Newcastle-Ottawa Scale (NOS) for cohort studies and Cochrane Risk-of-Bias tool (RoB 2) for randomized controlled trials. For NOS assessments, the considered domains included representativeness of the exposed cohort, selection of the non-exposed cohort, ascertainment of exposure, demonstration that the outcome of interest was not present at the start of the study, comparability of cohorts, assessment of outcomes, and adequate follow-up duration. The domains considered for RoB 2 assessments included the randomization process, deviations from intended interventions, missing outcome data, measurement of outcome, and selection of the reported result. Discrepancies were resolved by consensus involving two additional independent reviewers (J.S. and J.M.).

## Results

**Fig. 1:**
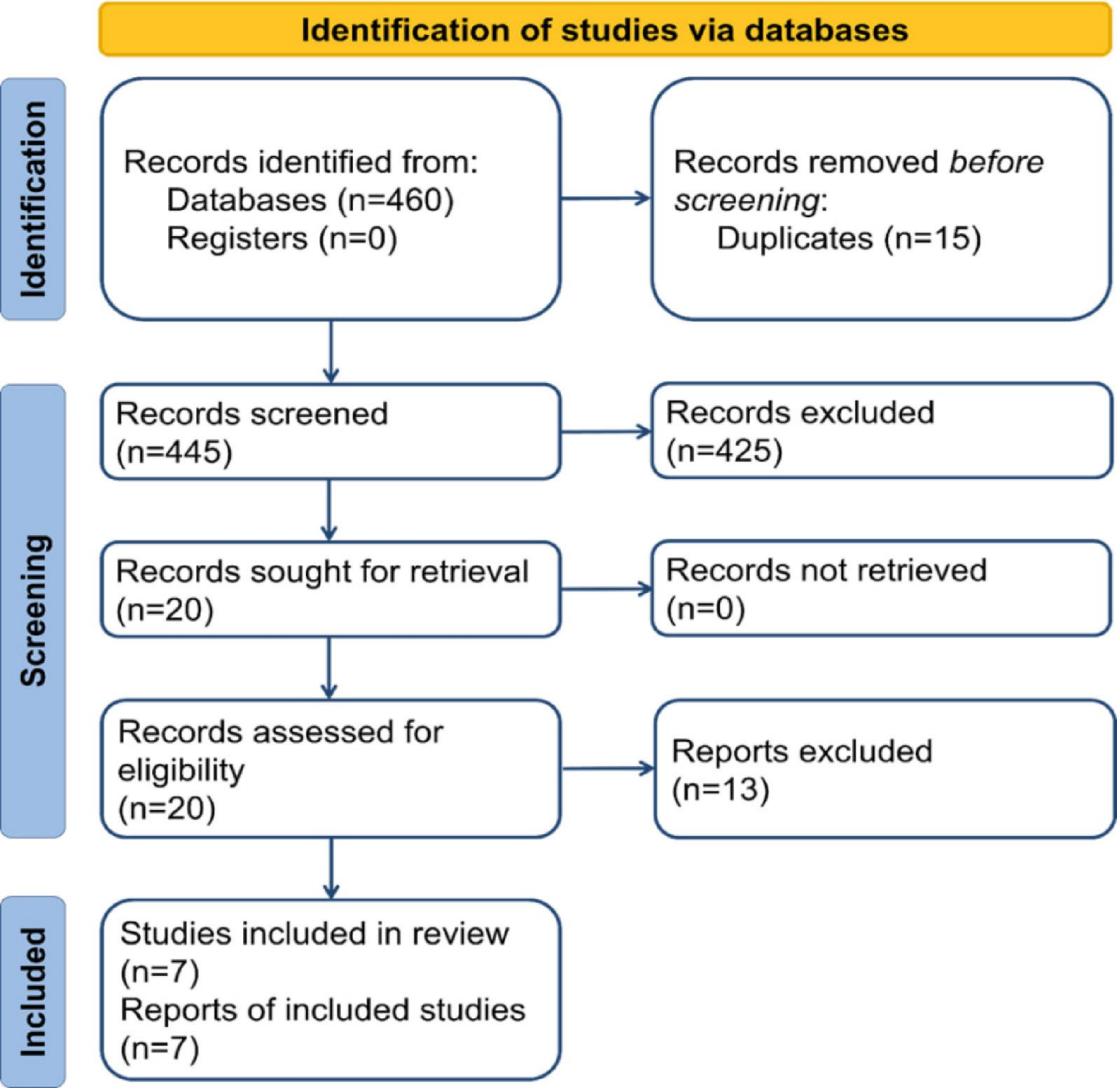
PRISMA flow diagram showing the selection process for studies included in the systematic review and meta-analysis of capitolunate fusion (CLF) versus three- or four-corner fusion (3CF/4CF)

The search yielded 459 articles that were screened. After removing duplicates, screening, and performing a full-text review, seven articles remained (Fig. 1). Six studies were retrospective cohort studies, and one was a randomized controlled trial. A total of 320 patients were included, including 149 with CLF, 164 with 4CF, and only seven underwent three-corner fusion, which precluded meaningful statistical comparison for this subgroup. Table 1 summarizes the demographic characteristics of the included study participants.

**Table 1:** Patient demographics of included studies. Demographic and baseline data for patients undergoing capitolunate fusion (CLF) compared with three- and four-corner fusion (3CF/4CF). Values are presented as mean (SD) or percentage unless otherwise indicated

Study	Intervention	Sample size	Wrist pathology	Procedure laterality right, %	Mean age, yrs	Males, %	Smoking hx, %
Duraku et al. [20]	CLF	34	SNAC, SLAC	77%	60 (11)	76%	9%
	4CF	29		52%	58 (10)	65%	27%
Elshahhat et al. [15]	CLF	31	SNAC	68%	31.5 (5.3)	87%	NR
	4CF	34		68%	35.6 (9.5)	94%	NR
Gaston et al. [18]	CLF	16	SLAC	NR	57 (10.1)	88%	42%
	4CF	18		NR	48 (14.4)	81%	44%
Kurucan et al. [19]	CLF	20	SNAC, SLAC	65%	60 (2.2)	90%	50%
	4CF	31		61%	62 (2.1)	90%	45%
Politikou et al. [24]	CLF	5	SNAC, SLAC, SCAC	NR	50.8 (11)	25%	NR
	3CF	7		NR			NR
Porto et al. [23]	CLF	11	SNAC	67%	40.54	82%	NR
	4CF	20		60%	46.5	85%	NR
Schriever et al. [5]	CLF	32	SNAC, SLAC	NR	60 (9.3)	78%	34%
	4CF	32		NR		91%	19%

Numbers in parentheses represent standard deviation. NR, not reported

Activity level and occupational demand were reported using study-specific definitions and are presented descriptively; no standardized classification or comparative analysis was performed

The mean age of the patients across the included studies ranged from 32 to 62 years. Patients undergoing 4CF tended to be older (mean age range: 36–62 years) than those receiving CLF (32–60 years). One cohort [15] featured distinctly younger patients in both arms, likely reflecting a younger high-demand population. Overall, male patients predominated across all cohorts, representing 65–94% of the population, whereas females comprised 6–35%.

Laterality data were inconsistently reported but suggested a slight predominance of right-sided surgeries across both procedures, typically ranging from 60 to 77%. In the four studies that reported this, left-sided surgeries ranged from 23 to 48%.

The etiology included scapholunate advanced collapse (SLAC), scaphoid nonunion advanced collapse (SNAC), or both. Where available, most patients had mixed etiologies (SLAC and SNAC or other indications). Notably, Gaston (2009) and Elshahhat (2024) reported purely SLAC- or SNAC-related pathology, while Kurucan (2024) and Schriever (2024) reported mixed or unspecified etiologies [5, 15, 18, 19].

The reporting of the SNAC stage varied across studies. Two studies explicitly reported SNAC stage distribution, including one study with both Stage II and Stage III wrists [15, 20] and one study limited to Stage III disease. [23] Several other studies grouped SNAC and SLAC wrists together or reported stages descriptively without stratified outcome data, precluding stage-based comparative analyses. 

Smoking status has been variably reported in the literature. Among the seven studies, only four had available data, among which the proportion of patients with a positive smoking history ranged from 9% (Duraku, CLF arm) to 50% (Kurucan, CLF arm), suggesting potential confounding for fusion outcomes [5, 18–20].

Patient occupation and activity levels were reported in several included studies; however, the reporting formats and definitions varied substantially. Some studies categorized patients using qualitative descriptors such as ‘high demand’ or ‘manual labor,’ whereas others reported graded activity levels or employment status. Due to this heterogeneity in categorization and the absence of standardized definitions, a meaningful comparison or quantitative synthesis of activity levels across studies was not feasible. 

Pre- and post-intervention VAS scores were reported in 4 studies. The average pre-intervention VAS score was lower for the CLF group (5.49, *n* = 80) than for the 3CF/4CF group (5.85, *n* = 88). The average post-intervention VAS score for CLF (2.04, *n* = 79) was higher than that for 3CF/4CF (1.88, *n* = 88). The pooled standard mean difference comparing CLF to 3CF/4CF was 0.34 (confidence interval [CI] − 0.004–0.69, *p* = 0.053). Although this result may indicate a tendency toward lower pain scores in the four-corner fusion cohort, the magnitude of the observed difference did not surpass the established minimal clinically important difference (MCID) thresholds for VAS in upper extremity conditions, which typically range from approximately 1.0 to 2.0 points. Therefore, this borderline finding is unlikely to reflect a clinically significant difference and should be interpreted cautiously.

No significant differences were found in the pre- to post-intervention changes between the CLF and 3CF/4CF groups for PRWE, grip strength, DASH score, and ROM (flexion, extension, pronation, supination, ulnar deviation, and radial deviation). For the evaluation of complications following surgery, no significant differences were found between the CLF and 3CF/4CF groups in terms of the occurrence of any adverse event, reoperation rate, and nonunion rate. The significance of the *p*-values was consistent across the different within-group correlation estimates (*r* = 0.3, 0.5, and 0.7).

**Fig. 2:**
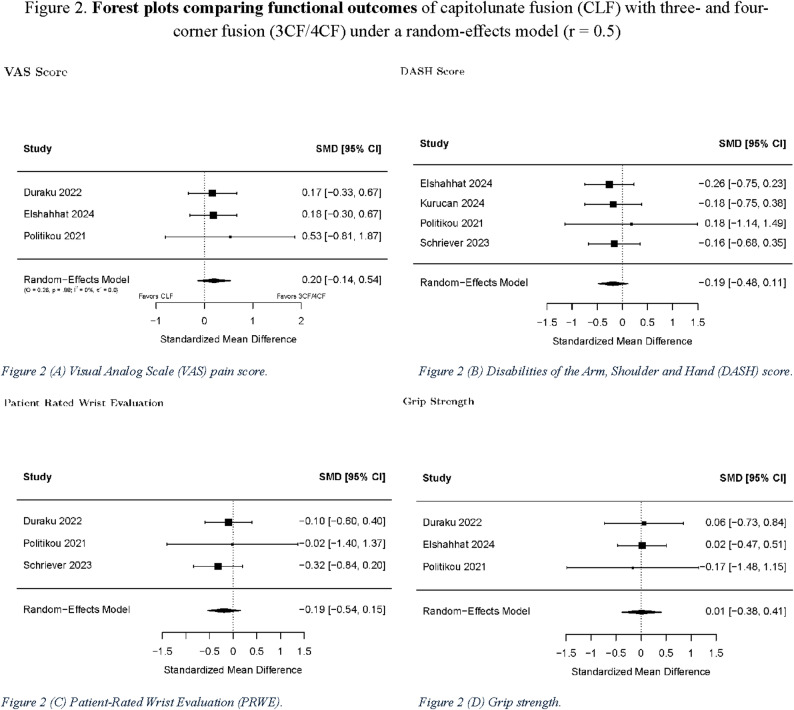
Forest plots comparing functional outcomes of capitolunate fusion (CLF) with three- and four-corner fusion (3CF/4CF) under a random-effects model (*r* = 0.5): **A** visual analog scale (VAS) pain score, **B** disabilities of the arm, shoulder and hand (DASH) score, **C** patient-rated wrist evaluation (PRWE), **D** grip strengthBlack squares represent standardized mean differences (SMDs); horizontal lines indicate 95% confidence intervals; black diamonds represent pooled effects. Positive values favor CLF.

**Fig. 3:**
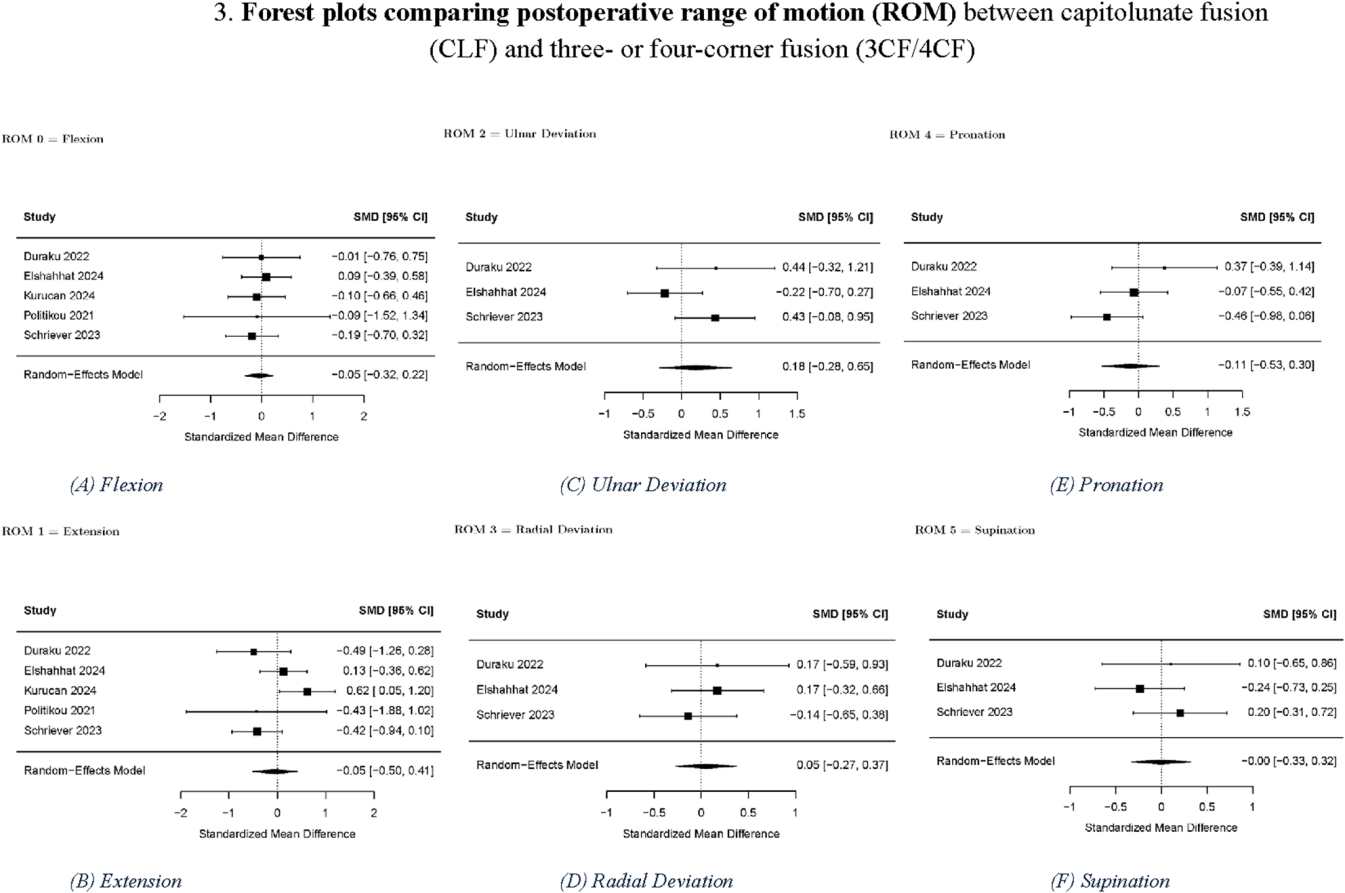
Forest plots comparing postoperative range of motion (ROM) between capitolunate fusion (CLF) and three- or four-corner fusion (3CF/4CF): **A** flexion, **B** extension, **C** ulnar deviation, D radial deviation, **E** pronation, **F** supinationBlack squares represent standardized mean differences (SMDs); horizontal lines indicate 95% confidence intervals; black diamonds represent pooled effects. Positive values favor CLF.

**Fig. 4:**
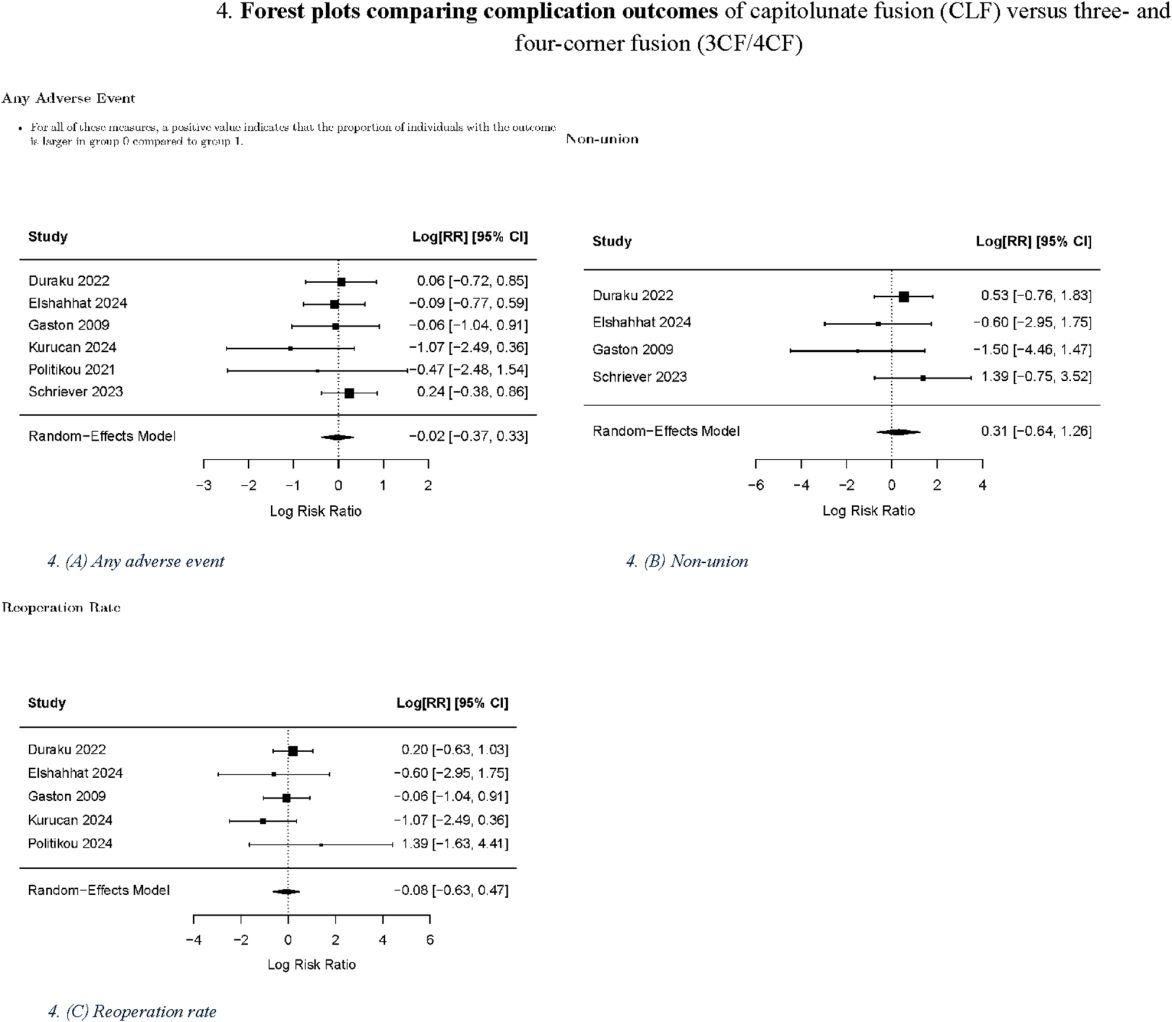
Forest plots comparing complication outcomes of capitolunate fusion (CLF) versus three- and four-corner fusion (3CF/4CF): **A** any adverse event, **B** nonunion rate, **C** reoperation rateBlack squares represent standardized mean differences (SMDs); horizontal lines indicate 95% confidence intervals; black diamonds represent pooled effects. Positive values favor CLF.

**Fig. 5:**
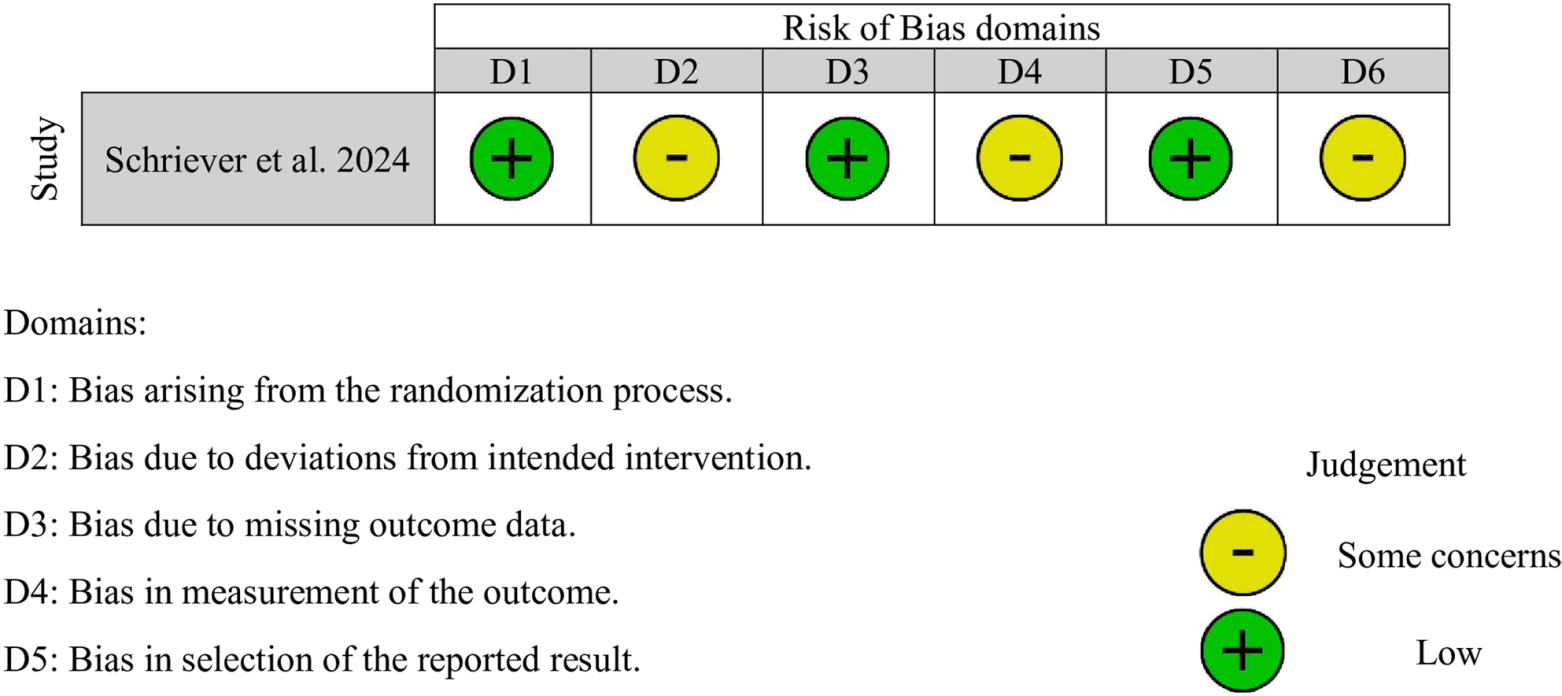
Cochrane RoB 2 risk-of-bias assessment for the randomized controlled trial. Evaluation of randomization process, deviations from intended interventions, missing outcome data, measurement of outcomes, and reporting bias.

Table 2 summarizes the pooled results for r = 0.5, and Figs. 2, 3 and 4 present the corresponding forest plots for each functional and complication outcome. Table 3. Provides a summary of the results concerning the various domains of study quality adapted from the Newcastle–Ottawa Scale. Each study was assessed based on eight criteria and categorized into three domains: selection of study groups, comparability of groups, and assessment of outcomes of interest. Nine stars were considered to be indicative of the highest quality. Additionally, Fig. 5. Presents the results of the RoB 2 risk assessment tool for evaluating bias in shriever et al. [5].

**Table 2:** Summary of meta-analytic results. Pooled standardized mean differences (SMDs) or risk ratios (RRs) comparing capitolunate fusion (CLF) and three- or four-corner fusion (3CF/4CF) for each outcome measure under a random-effects model

Outcome	Studies (*n*)	Heterogeneity (I^2^, Q, *p*)	Pooled effect [95% CI]	*p*-value	Interpretation
Continuous outcomes	SMD				
VAS score	4	Low (I^2^ = 14.5%, Q = 3.57, *p* = 0.31)	0.34 [– 0.004, 0.69]	0.053	Borderline nonsignificant favoring 3CF/4CF
PRWE	3	Low (I^2^ = 0%, Q = 0.43, *p* = 0.81)	– 0.19 [– 0.54, 0.15]	0.27	No significant difference
Grip strength	3	Low (I^2^ = 0%, Q = 0.08, *p* = 0.96)	0.01 [– 0.38, 0.41]	0.95	No significant difference
DASH score	4	Low (I^2^ = 0%, Q = 0.38, *p* = 0.94)	– 0.19 [– 0.48, 0.11]	0.21	No significant difference
Flexion–Extension arc motion	5	Moderate (I^2^ = 59.5%, Q = 9.76, *p* = 0.04)	– 0.10 [– 0.57, 0.36]	0.67	No significant difference
Pronation–Supination arc motion	3	Low (I^2^ ≈ 0%, Q = 2.66, *p* = 0.26)	– 0.17 [– 0.49, 0.15]	0.30	No significant difference
Radial–Ulnar arc motion	3	Moderate (I^2^ = 42.8%, Q = 3.47, *p* = 0.18)	0.21 [– 0.23, 0.65]	0.35	No significant difference

Outcome	Studies (*n*)	Heterogeneity (I^2^, Q, *p*)	Pooled effect [95% CI]	*p*-value	Interpretation
Adverse events	RR				
Any adverse event	6	Low (I^2^ = 0%, Q = 3.04, *p* = 0.69)	RR = 0.98 [0.69, 1.39]	0.91	No significant difference
Reoperation rate	6	Low (I^2^ = 0%, Q = 3.36, *p* = 0.64)	RR = 0.92 [0.54, 1.58]	0.77	No significant difference
Nonunion rate	4	Low (I^2^ = 0%, Q = 3.09, *p* = 0.37)	RR = 1.36 [0.53, 3.52]	0.52	No significant difference

SMD, standardized mean difference; RR, risk ratio

**Table 3:** Newcastle–Ottawa scale (NOS) risk-of-bias assessment. Quality ratings for included retrospective and prospective cohort studies based on selection, comparability, and outcome domains

Study ID	Selection				Comparability	Outcome			Total
	Representativeness of the exposed cohort	Selection of the non-exposed cohort	Ascertainment of exposure	Demonstration that outcome of interest was not present at start of study	Comparability of cohorts on the basis of the design or analysis	Assessment of outcome	Was follow-up long enough for outcomes to occur	Adequacy of follow up of cohorts	
Gaston [18]	*	*	*			*	*		5
Porto [23]	*	*	*	*	*	*	*	*	8
Politikou [24]		*	*	*		*	*		5
Duraku, [20]	*	*	*	*	*	*	*	*	8
Elshahat [15]	*	*	*	*		*	*	*	7
Kurucan [19]	*	*	*	*	*	*		*	7

**Table 4:** Clinical implications of capitolunate fusion versus four-corner fusion for advanced wrist collapse.

Clinical domain	Evidence from this review	Practical implication	Caveats
Pain relief	No statistically significant difference in VAS	Either procedure may provide comparable pain relief	Borderline trend favoring 4CF; difference below MCID
Functional outcomes	No significant differences in DASH or PRWE	Functional recovery appears similar between procedures	Heterogeneity in outcome reporting
Range of motion	No consistent differences across motion planes	Motion preservation is comparable overall	ROM assessed by individual planes
Union rates	No significant difference in nonunion rates	Similar likelihood of achieving union	Based on mostly retrospective data
Complications	Comparable overall complication and reoperation rates	Risk profiles appear similar	Limited power to detect rare events
Procedure selection	Broadly comparable outcomes	Choice may be individualized based on patient anatomy, demands, and surgeon experience	No direct comparison of technical complexity

## Discussion

### Principal findings

Capitolunate fusion (CLF) has emerged as a motion-preserving surgical option for managing advanced wrist osteoarthritis, particularly in cases of scapholunate advanced collapse (SLAC) and scaphoid nonunion advanced collapse (SNAC). The largest study to date included six retrospective studies and one randomized controlled trial. The majority of patients were male and had mixed SLAC/SNAC etiologies. Given that most of the included studies were retrospective with heterogeneous fixation methods and follow-up durations, future prospective multicenter trials are warranted to delineate whether the technical simplicity of capitolunate fusion translates into improved cost-effectiveness and faster rehabilitation compared to four-corner fusion.

### Outcomes

Interestingly, our results align with those of the largest previous systematic review [6], which reported that capitolunate fusion is a viable option for managing SLAC and SNAC wrists, with complication rates comparable to those of 4CF. Furthermore, some evidence suggests that CLF may offer a modest advantage in wrist flexion and, in certain series, greater grip strength than 4CF [15, 18]. Our results from a large cohort of 320 patients confirm that CLF achieves significant pain relief and functional improvement, with most patients returning to work and reporting high satisfaction, with no clinically meaningful difference in the range of motion, grip strength, or patient-reported outcomes at 12 months. These results are in line with those of a recent meta-analysis and systematic review, further supporting our claim that both CLF and 4CF are viable options for SLAC/SNAC Stage III disease owing to their similar pain reduction, functional scores, and complication rates [21]. Accordingly, while no statistically significant differences were identified, this finding indicates broadly comparable outcomes between the two procedures, rather than definitive equivalence.

### Complications

Complication rates for CLF are similar to those for 4CF, with nonunion rates typically below 5% and reoperation rates ranging from 10 to 14% [6, 7, 11, 20]. Hardware migration and the need for screw removal are more frequently reported with CLF, whereas 4CF may be associated with subsequent pisotriquetral arthritis and a slightly higher risk of radiolunate arthritis [18]. The choice of fixation method did not appear to significantly impact union rates or functional outcomes in either procedure [22]. Across the included studies, a range of osteosynthesis techniques were used for three- and four-corner fusions, including dorsal plates (e.g., spider or circular plates), headless or compression screws, K-wires, staples, and combined fixation methods. However, most studies reported union outcomes at the procedure level without stratifying the results by specific fixation technique. Consequently, a meaningful comparison of nonunion rates by osteosynthesis method across studies was not feasible. Only one comparative study reported fixation-specific nonunion within the four-corner fusion group, with nonunions observed in cases using a dorsal plate construct, whereas no nonunions were reported with screw or staple fixations. This finding should be interpreted cautiously, given the limited sample size and lack of corroboration between studies [23]. In summary, in the largest systematic review and meta-analysis to date, we found that capitolunate arthrodesis is a viable alternative to four-corner fusion for advanced wrist collapse, with no statistically significant differences in pain relief, functional recovery, and union rates, which renders the choice between these procedures individualized, considering patient anatomy, functional demands, and surgeon experience. Technical considerations, such as operative complexity and efficiency, were not directly evaluated and therefore cannot be compared between procedures. Further high-quality, long-term comparative studies are needed to clarify the durability of these procedures, optimize patient selection, and better contextualize these findings for orthopaedic hand surgeons.

### Limitations

Only seven studies satisfied our inclusion criteria, of which six were retrospective cohort studies, which are inherently associated with an elevated risk of selection and reporting biases. Additionally, given the limited number of included studies, formal evaluation of publication bias using funnel plots or Egger’s test was not undertaken, as such methods are not recommended in small meta-analyses because of low statistical power. The limited cumulative sample size constrained the statistical power and generalizability of our findings. Incomplete and inconsistent reporting of several demographic and clinical variables was common across the included studies, which limited the feasibility of detailed subgroup analysis. Patient occupation and activity level were reported using heterogeneous, study-specific definitions of “high-demand” work and non-uniform categorization of activity intensity, precluding meaningful comparison or stratified analysis. Regarding range of motion, while a composite range-of-motion outcome could reduce fragmentation, variability in reporting and the lack of a validated composite metric limited its feasibility in this analysis. Variability was also observed in patient demographics, surgical techniques, fixation methods, and follow-up duration; however, statistical heterogeneity across pooled analyses was generally low to moderate and is unlikely to have materially influenced the robustness or direction of the main conclusions of this review. SLAC and SNAC wrists were pooled in the primary analyses because of inconsistent and incomplete reporting of disease stages across studies. Specifically, SNAC staging has been reported variably, with only a limited number of studies providing stage-specific data. This variability restricted the ability to perform stratified analyses and diminished clinical precision when differentiating outcomes between Stage II and Stage III diseases. Data for three outcomes were extracted from graphical box plots using digital plot extraction software (PlotDigitizer), including the PRWE and DASH scores from Schriever et al. and Duraku et al. The extracted variables included flexion, extension, radial deviation, ulnar deviation, and pronation–supination; this approach may have introduced minor measurement errors [5, 20] Several outcomes were reported as medians with interquartile ranges and were converted to means and standard deviations under the assumption of approximate normality, which may introduce estimation errors and should be considered when interpreting the results. Finally, potential publication bias cannot be excluded because only English-language studies were included. Future large-scale multicenter randomized controlled trials with standardized reporting and long-term follow-up are warranted to validate these findings and optimize patient selection for each technique.

## Supplementary Information

Below is the link to the electronic supplementary material.

Supplementary Material 1. Full Forest Plots Tables S1–S13 depict forest plots of the standardized mean difference or risk ratio, including analysis by study and a random effects model, for each of the following measures: VAS pain score, PRWE, grip strength, DASH score, flexion, extension, ulnar deviation, radial deviation, pronation, supination, any adverse event, reoperation rate, and nonunion.

Supplementary Material 2. Full Search Strategies This file contains the complete search strategies used in PubMed, EMBASE, and Web of Science, including all keywords, MeSH terms, Boolean operators, and filters applied in the systematic search conducted on December 27, 2024.

## Data Availability

The dataset used in this study is available from the corresponding author on reasonable request.
