# Supplementary material for: Capitolunate arthrodesis versus four-corner fusion for advanced wrist collapse: a systematic review and meta-analysis
Source: J Orthop Surg Res. 2026 Mar 13;21:268. doi: 10.1186/s13018-026-06747-x (PMC13097632; doi:10.1186/s13018-026-06747-x)
Supplement: Supplementary file 1 — Supplementary Material 1. Full Forest Plots Tables S1–S13 depict forest plots of the standardized mean difference or risk ratio, including analysis by study and a random effects model, for each of the following measures: VAS pain score, PRWE, grip strength, DASH score, flexion, extension, ulnar deviation, radial deviation, pronation, supination, any adverse event, reoperation rate, and nonunion. [file 13018_2026_6747_MOESM1_ESM.pdf]

Capitolunate Arthrodesis as an Efficient, Motion-Preserving Alternative to Corner Fusions for Advanced Wrist Collapse: A Systematic Review and Meta-Analysis

3-30-25

- Figures generated with correlation estimate = 0.5
- Sensitivity analysis shows no change in significance across ro = 0.3, 0.5, 0.7

VAS Score

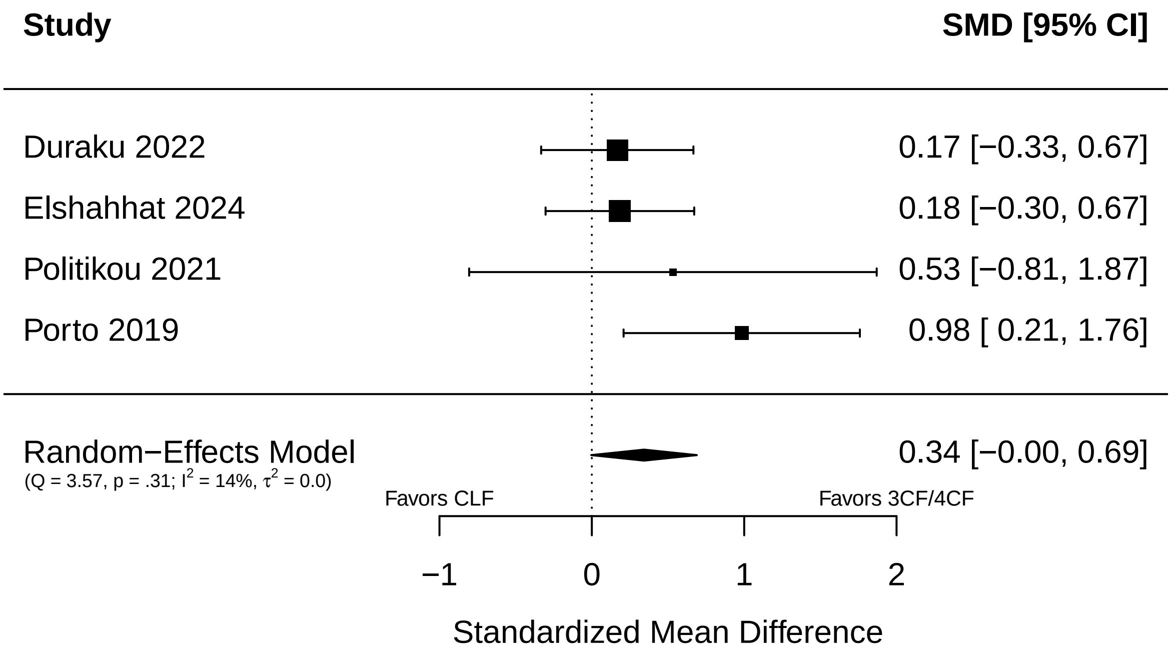


```
##          StudyID Group0_mean_change Group0_sd_change Group0_N_change
## 1    Duraku 2022            -2.3725         3.548304              33
## 2 Elshahhat 2024            -5.0200         1.571623              31
## 3 Politikou 2021            -4.5000         1.534318               4
## 4     Porto 2019            -1.8000         1.178983              11
##   Group1_mean_change Group1_sd_change Group1_N_change
## 1            -2.9735        3.5592193              29
## 2            -5.3300        1.7435596              34
## 3            -5.6000        2.0332748               5
## 4            -2.7000        0.6928203              20
```

- Black square is SMD. Size of black square indicates weight (# of patients)
- CIs
- Black diamond: pooled (combined) estimate under the RE model.

```
##
## Random-Effects Model (k = 4; tau^2 estimator: REML)
##
##   logLik  deviance       AIC       BIC      AICc
##  -1.6162    3.2323    7.2323    5.4295   19.2323
##
## tau^2 (estimated amount of total heterogeneity): 0.0191 (SE = 0.0995)
## tau (square root of estimated tau^2 value):      0.1383
## I^2 (total heterogeneity / total variability):   14.46%
## H^2 (total variability / sampling variability):  1.17
##
## Test for Heterogeneity:
## Q(df = 3) = 3.5693, p-val = 0.3119
##
## Model Results:
##
## estimate      se    zval    pval    ci.lb   ci.ub
##   0.3426  0.1768  1.9371  0.0527  -0.0040  0.6892  .
##
## ---
## Signif. codes:  0 '***' 0.001 '**' 0.01 '*' 0.05 '.' 0.1 ' ' 1
```

- k = 4 studies
- estimate, se, **p-value**, CI
- Q statistic: Test for Heterogeneity. No statistically significant heterogeneity across the studies.
- tau^2: the between-study variance, aka variance of the true effect sizes across studies. Estimated using the Restricted Maximum Likelihood (REML) methods. tau^2 = 0 => minimal or no detectable heterogeneity in data set
- logLike / AIC / BIC: goodness of fit and model comparison stats, not so relevant here
- I^2 describes the proportion of total variance in the observed effects that is due to true heterogeneity rather than sampling error. I^2 = 0%, indicating essentially no heterogeneity among studies.
- H^2 is another measure of total variability vs. sampling variability (also 1.0 here, meaning no excess heterogeneity beyond chance).
- If considerable heterogeneity, we should do moderator analysis.
- Do we find statistically significant difference in pain improvement (VAS score) between CLF and 3CF/4CF?

Patient Rated Wrist Evaluation

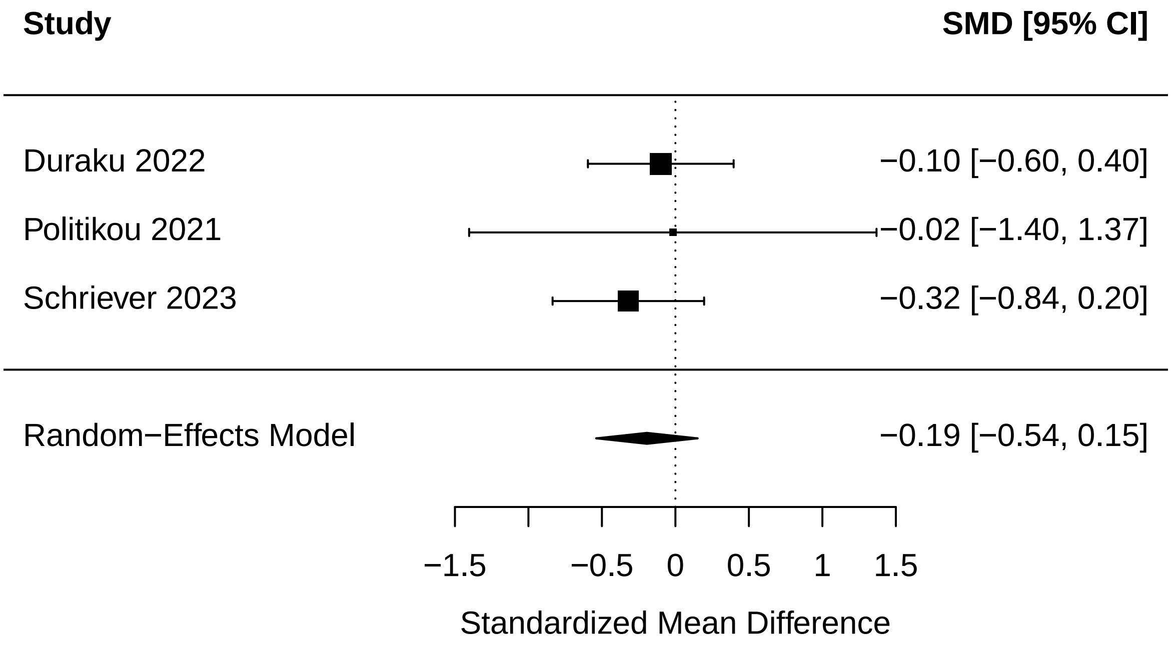


```
##
## Random-Effects Model (k = 3; tau^2 estimator: REML)
##
##   logLik  deviance       AIC       BIC      AICc
##  -0.1818    0.3636    4.3636    1.7499   16.3636
##
## tau^2 (estimated amount of total heterogeneity): 0 (SE = 0.0925)
## tau (square root of estimated tau^2 value):      0
## I^2 (total heterogeneity / total variability):   0.00%
## H^2 (total variability / sampling variability):  1.00
##
## Test for Heterogeneity:
## Q(df = 2) = 0.4316, p-val = 0.8059
##
## Model Results:
##
## estimate      se     zval    pval    ci.lb   ci.ub
##  -0.1939  0.1765  -1.0984  0.2720  -0.5399  0.1521
##
## ---
## Signif. codes:  0 '***' 0.001 '**' 0.01 '*' 0.05 '.' 0.1 ' ' 1
```

Grip Strength

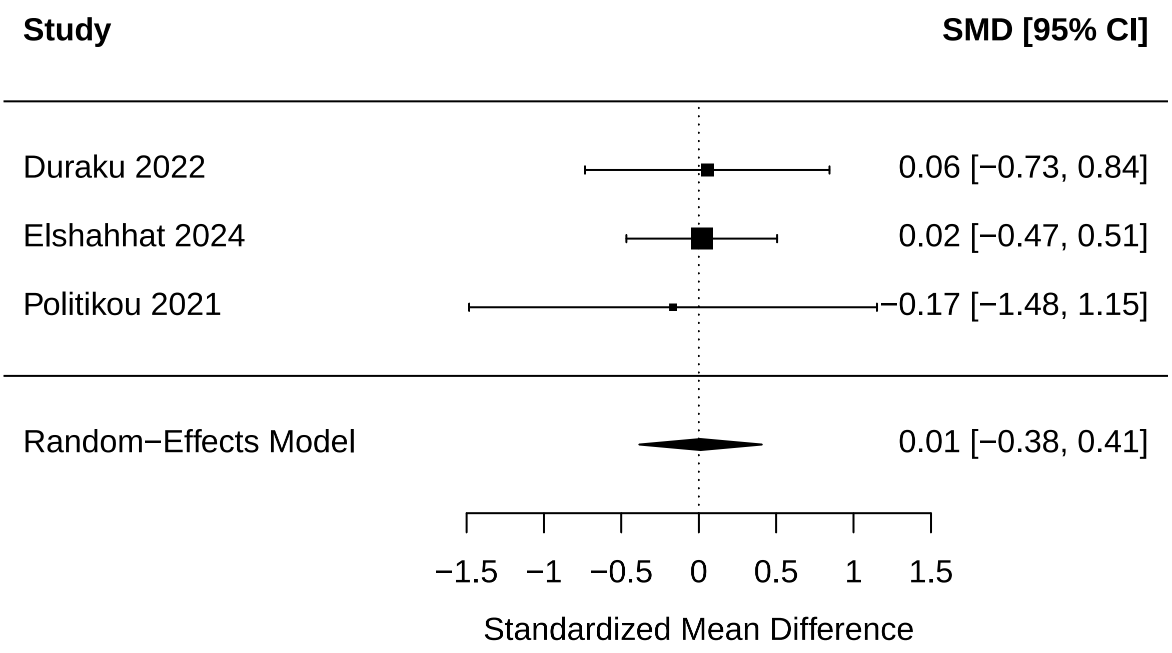


```
##
## Random-Effects Model (k = 3; tau^2 estimator: REML)
##
##   logLik  deviance       AIC       BIC      AICc
##  -0.2316    0.4632    4.4632    1.8495   16.4632
##
## tau^2 (estimated amount of total heterogeneity): 0 (SE = 0.1461)
## tau (square root of estimated tau^2 value):      0
## I^2 (total heterogeneity / total variability):   0.00%
## H^2 (total variability / sampling variability):  1.00
##
## Test for Heterogeneity:
## Q(df = 2) = 0.0826, p-val = 0.9596
##
## Model Results:
##
## estimate      se    zval    pval    ci.lb   ci.ub
##   0.0118  0.2017  0.0583  0.9535  -0.3835  0.4070
##
## ---
## Signif. codes:  0 '***' 0.001 '**' 0.01 '*' 0.05 '.' 0.1 ' ' 1

##
```

```
##          StudyID Group0_mean_change Group0_sd_change Group0_N_change
## 1    Duraku 2022               0.37         8.824799              11
## 2 Elshahhat 2024              10.30         5.230679              31
## 3 Politikou 2021              -4.00        10.364683               4
##   Group1_mean_change Group1_sd_change Group1_N_change      yi     vi
## 1             -0.315        14.000000              14  0.0551 0.1624
## 2             10.200         4.866210              34  0.0196 0.0617
## 3             -2.300         8.010363               5 -0.1660 0.4515
```

DASH Score

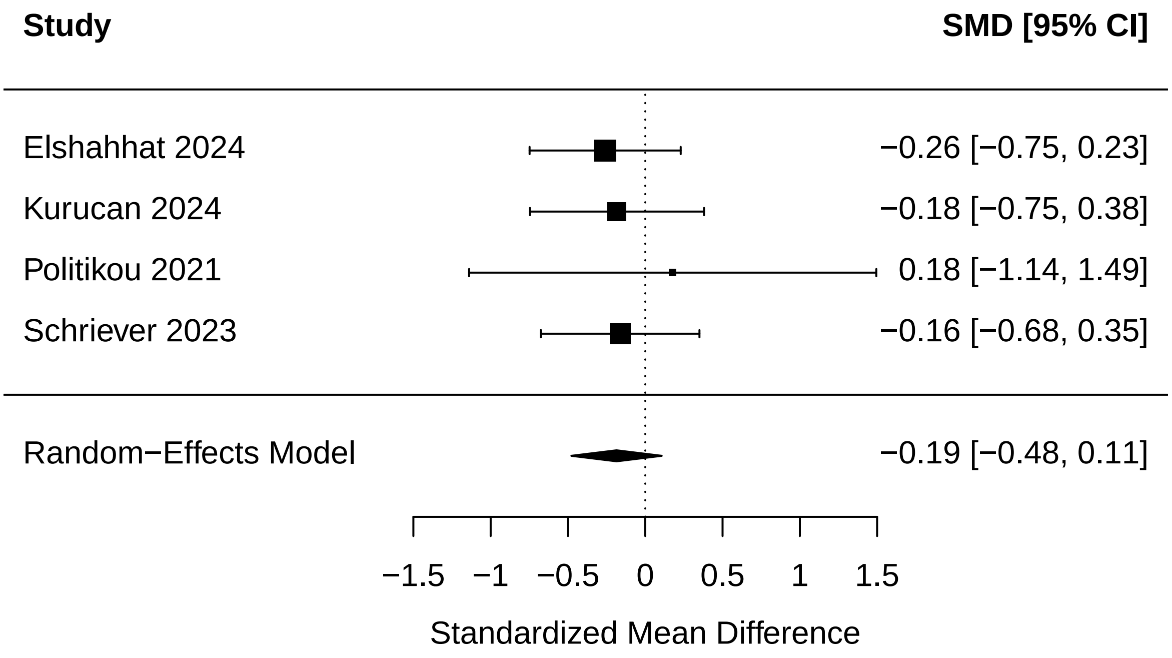


```
##
## Random-Effects Model (k = 4; tau^2 estimator: REML)
##
##   logLik  deviance       AIC       BIC      AICc
##   0.2127   -0.4253    3.5747    1.7719   15.5747
##
## tau^2 (estimated amount of total heterogeneity): 0 (SE = 0.0696)
## tau (square root of estimated tau^2 value):      0
## I^2 (total heterogeneity / total variability):   0.00%
## H^2 (total variability / sampling variability):  1.00
##
## Test for Heterogeneity:
## Q(df = 3) = 0.3876, p-val = 0.9428
##
## Model Results:
##
## estimate      se     zval    pval    ci.lb   ci.ub
##  -0.1859  0.1491  -1.2470  0.2124  -0.4781  0.1063
##
## ---
## Signif. codes:  0 '***' 0.001 '**' 0.01 '*' 0.05 '.' 0.1 ' ' 1
```

Reoperation Rate

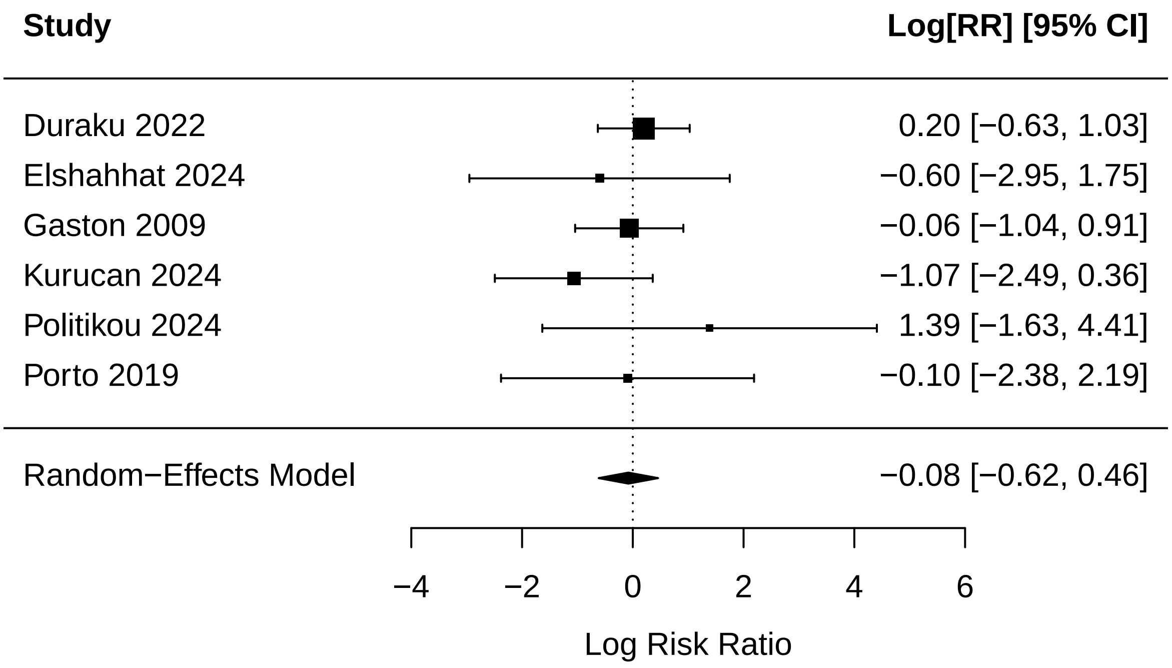


```
##
## Random-Effects Model (k = 6; tau^2 estimator: REML)
##
##   logLik  deviance       AIC       BIC      AICc
##  -5.5692   11.1384   15.1384   14.3573   21.1384
##
## tau^2 (estimated amount of total heterogeneity): 0 (SE = 0.2563)
## tau (square root of estimated tau^2 value):      0
## I^2 (total heterogeneity / total variability):   0.00%
## H^2 (total variability / sampling variability):  1.00
##
## Test for Heterogeneity:
## Q(df = 5) = 3.3632, p-val = 0.6442
##
## Model Results:
##
## estimate      se     zval    pval    ci.lb   ci.ub
##  -0.0805  0.2737  -0.2941  0.7686  -0.6169  0.4559
##
## ---
## Signif. codes:  0 '***' 0.001 '**' 0.01 '*' 0.05 '.' 0.1 ' ' 1
```

Any Adverse Event

- For all of these measures, a positive value indicates that the proportion of individuals with the outcome is larger in group 0 compared to group 1.

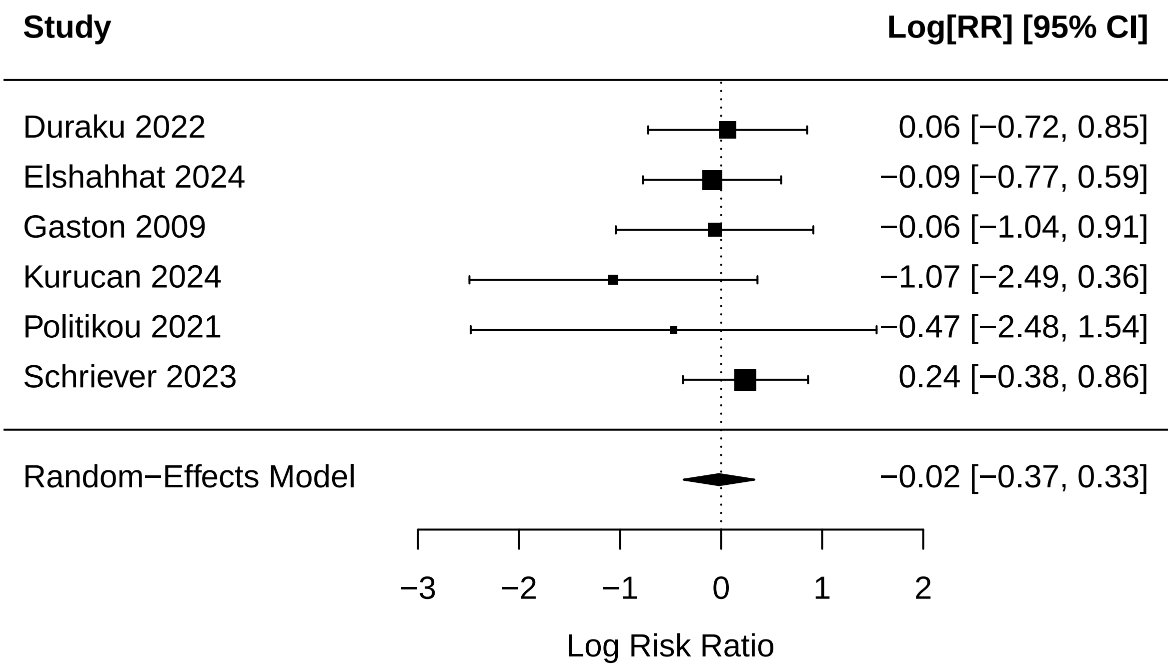


```
##
## Random-Effects Model (k = 6; tau^2 estimator: REML)
##
##   logLik  deviance       AIC       BIC      AICc
##  -2.8306    5.6612    9.6612    8.8801   15.6612
##
## tau^2 (estimated amount of total heterogeneity): 0 (SE = 0.1118)
## tau (square root of estimated tau^2 value):      0
## I^2 (total heterogeneity / total variability):   0.00%
## H^2 (total variability / sampling variability):  1.00
##
## Test for Heterogeneity:
## Q(df = 5) = 3.0362, p-val = 0.6944
##
## Model Results:
##
## estimate      se     zval    pval    ci.lb   ci.ub
##  -0.0207  0.1787  -0.1159  0.9077  -0.3710  0.3296
##
## ---
## Signif. codes:  0 '***' 0.001 '**' 0.01 '*' 0.05 '.' 0.1 ' ' 1
```

```
##
## Random-Effects Model (k = 6; tau^2 estimator: REML)
##
##   logLik  deviance       AIC       BIC      AICc
##  -2.8306    5.6612    9.6612    8.8801   15.6612
##
## tau^2 (estimated amount of total heterogeneity): 0 (SE = 0.1118)
## tau (square root of estimated tau^2 value):      0
## I^2 (total heterogeneity / total variability):   0.00%
## H^2 (total variability / sampling variability):  1.00
##
## Test for Heterogeneity:
## Q(df = 5) = 3.0362, p-val = 0.6944
##
## Model Results:
##
## estimate      se     zval    pval    ci.lb   ci.ub
##  -0.0207  0.1787  -0.1159  0.9077  -0.3710  0.3296
##
## ---
## Signif. codes:  0 '***' 0.001 '**' 0.01 '*' 0.05 '.' 0.1 ' ' 1
```

```
## pdf
##   2
```

Non-union

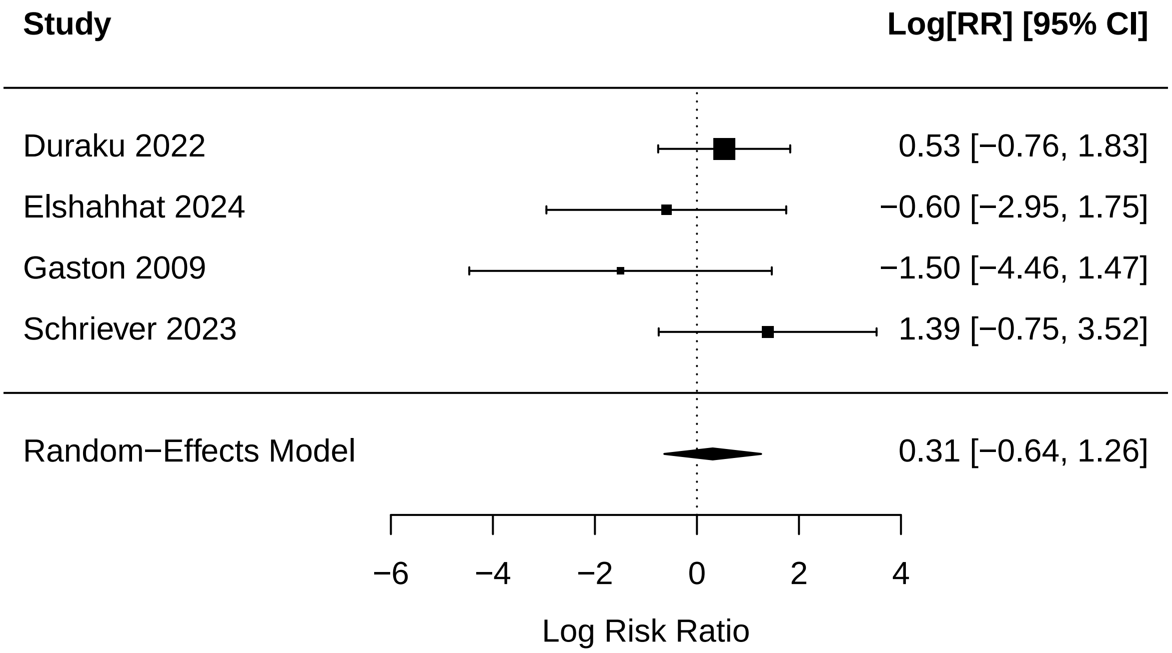


```
##
## Random-Effects Model (k = 4; tau^2 estimator: REML)
##
##   logLik  deviance       AIC       BIC      AICc
##  -4.6039    9.2078   13.2078   11.4050   25.2078
##
## tau^2 (estimated amount of total heterogeneity): 0.0000 (SE = 0.8253)
## tau (square root of estimated tau^2 value):      0.0010
## I^2 (total heterogeneity / total variability):   0.00%
## H^2 (total variability / sampling variability):  1.00
##
## Test for Heterogeneity:
## Q(df = 3) = 3.0961, p-val = 0.3771
##
## Model Results:
##
## estimate      se    zval    pval    ci.lb   ci.ub
##   0.3093  0.4841  0.6389  0.5229  -0.6395  1.2581
##
## ---
## Signif. codes:  0 '***' 0.001 '**' 0.01 '*' 0.05 '.' 0.1 ' ' 1

##
```

```
## Random-Effects Model (k = 4; tau^2 estimator: REML)
##
##   logLik  deviance       AIC       BIC      AICc
##  -4.6039    9.2078   13.2078   11.4050   25.2078
##
## tau^2 (estimated amount of total heterogeneity): 0.0000 (SE = 0.8253)
## tau (square root of estimated tau^2 value):      0.0010
## I^2 (total heterogeneity / total variability):   0.00%
## H^2 (total variability / sampling variability):  1.00
##
## Test for Heterogeneity:
## Q(df = 3) = 3.0961, p-val = 0.3771
##
## Model Results:
##
## estimate      se    zval    pval    ci.lb   ci.ub
##   0.3093  0.4841  0.6389  0.5229  -0.6395  1.2581
##
## ---
## Signif. codes:  0 '***' 0.001 '**' 0.01 '*' 0.05 '.' 0.1 ' ' 1
```

```
## pdf
##   2
```

ROM 0 = Flexion

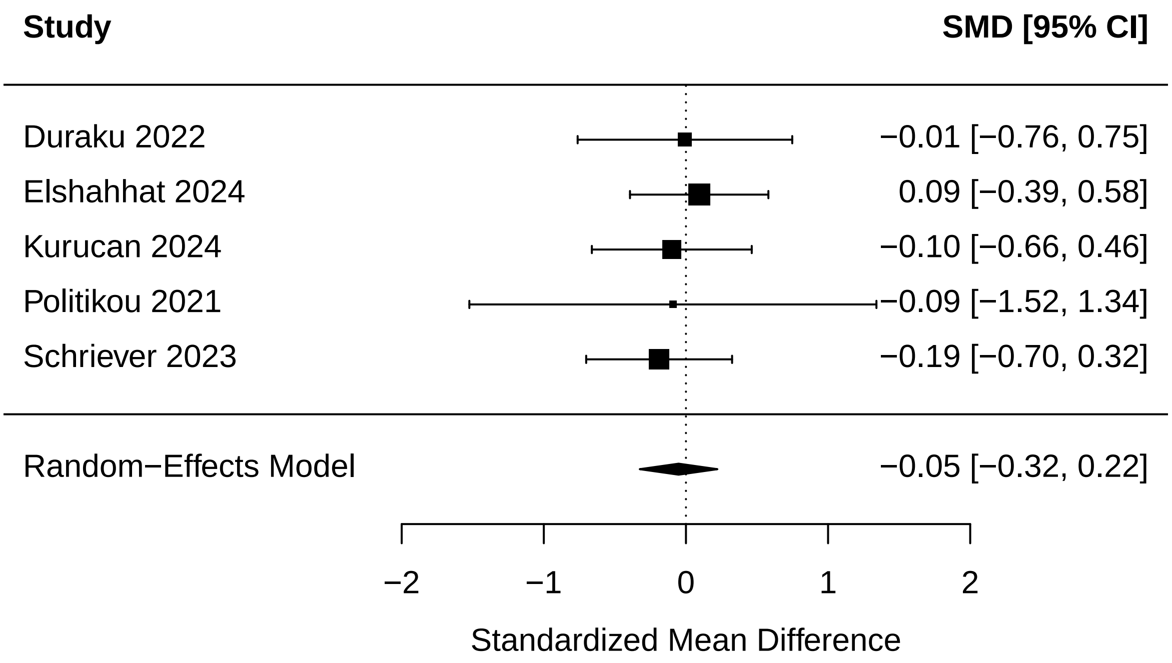


```
##
## Random-Effects Model (k = 5; tau^2 estimator: REML)
##
##   logLik  deviance       AIC       BIC      AICc
##   0.0776   -0.1552    3.8448    2.6174   15.8448
##
## tau^2 (estimated amount of total heterogeneity): 0 (SE = 0.0649)
## tau (square root of estimated tau^2 value):      0
## I^2 (total heterogeneity / total variability):   0.00%
## H^2 (total variability / sampling variability):  1.00
##
## Test for Heterogeneity:
## Q(df = 4) = 0.6575, p-val = 0.9565
##
## Model Results:
##
## estimate      se     zval    pval    ci.lb   ci.ub
##  -0.0518  0.1393  -0.3720  0.7099  -0.3248  0.2212
##
## ---
## Signif. codes:  0 '***' 0.001 '**' 0.01 '*' 0.05 '.' 0.1 ' ' 1

## pdf
##   2
```

ROM 1 = Extension

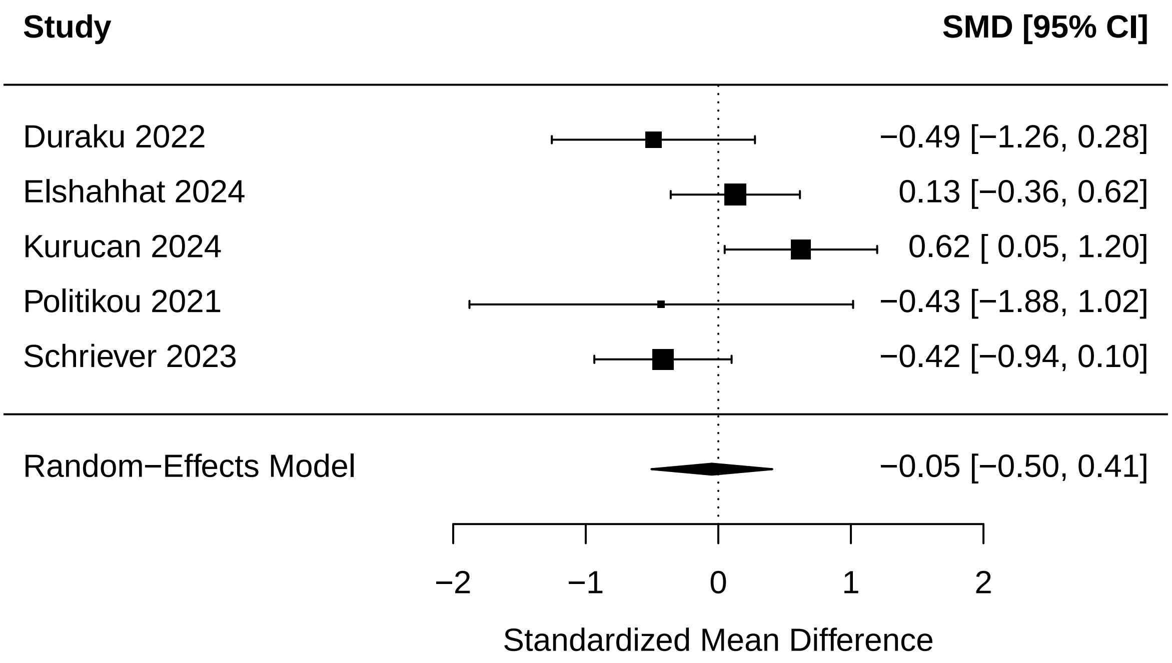


```
##
## Random-Effects Model (k = 5; tau^2 estimator: REML)
##
##   logLik  deviance       AIC       BIC      AICc
##  -3.0532    6.1064   10.1064    8.8790   22.1064
##
## tau^2 (estimated amount of total heterogeneity): 0.1473 (SE = 0.1871)
## tau (square root of estimated tau^2 value):      0.3838
## I^2 (total heterogeneity / total variability):   58.11%
## H^2 (total variability / sampling variability):  2.39
##
## Test for Heterogeneity:
## Q(df = 4) = 9.1755, p-val = 0.0569
##
## Model Results:
##
## estimate      se     zval    pval    ci.lb   ci.ub
##  -0.0485  0.2328  -0.2083  0.8350  -0.5048  0.4078
##
## ---
## Signif. codes:  0 '***' 0.001 '**' 0.01 '*' 0.05 '.' 0.1 ' ' 1
```

```
## pdf
##   2
```

ROM 2 = Ulnar Deviation

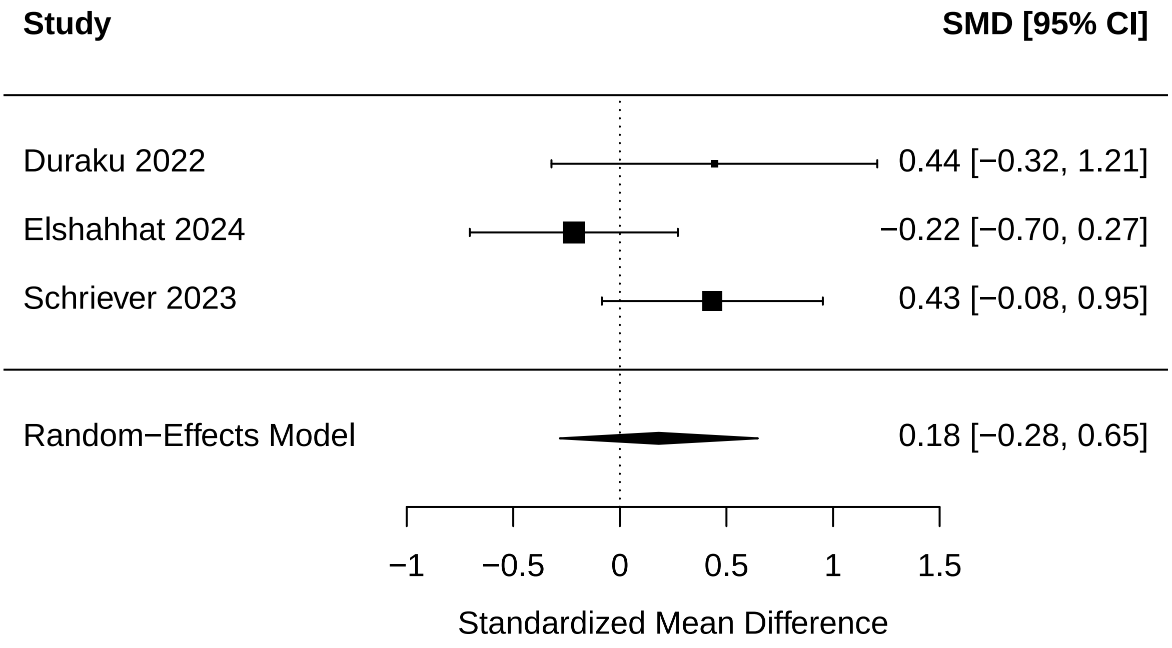


```
##
## Random-Effects Model (k = 3; tau^2 estimator: REML)
##
##   logLik  deviance       AIC       BIC      AICc
##  -0.9956    1.9911    5.9911    3.3774   17.9911
##
## tau^2 (estimated amount of total heterogeneity): 0.0821 (SE = 0.1698)
## tau (square root of estimated tau^2 value):      0.2865
## I^2 (total heterogeneity / total variability):   48.87%
## H^2 (total variability / sampling variability):  1.96
##
## Test for Heterogeneity:
## Q(df = 2) = 3.8791, p-val = 0.1438
##
## Model Results:
##
## estimate      se    zval    pval    ci.lb   ci.ub
##   0.1826  0.2371  0.7703  0.4411  -0.2821  0.6473
##
## ---
## Signif. codes:  0 '***' 0.001 '**' 0.01 '*' 0.05 '.' 0.1 ' ' 1
```

ROM 3 = Radial Deviation

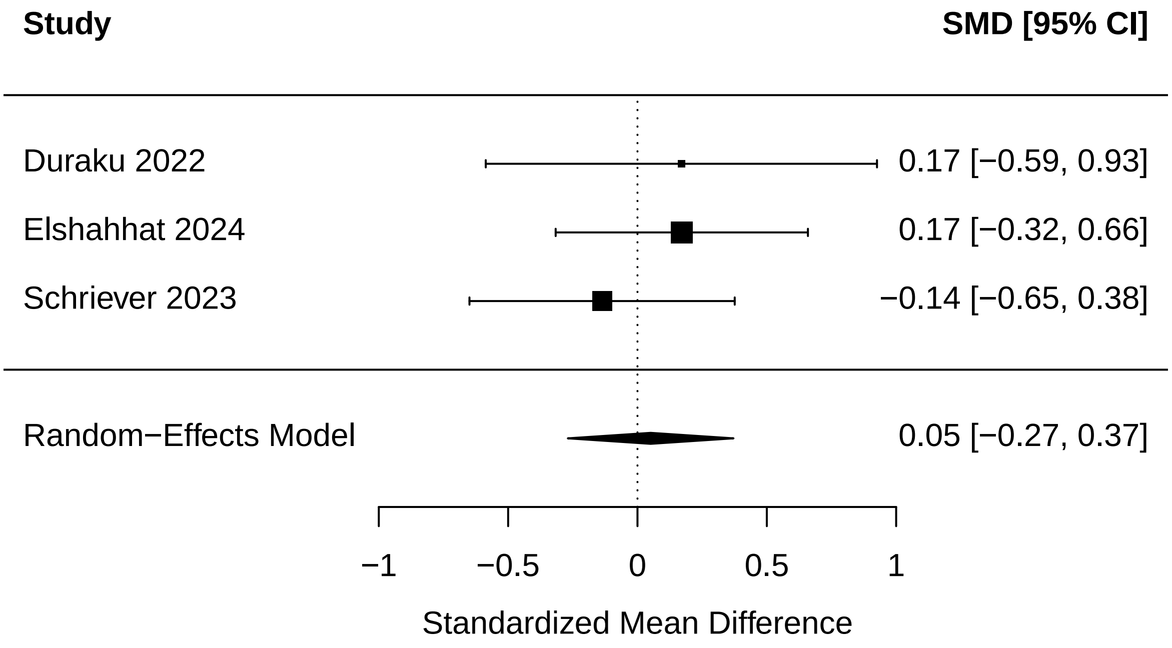


```
##
## Random-Effects Model (k = 3; tau^2 estimator: REML)
##
##   logLik  deviance       AIC       BIC      AICc
##   0.1618   -0.3236    3.6764    1.0627   15.6764
##
## tau^2 (estimated amount of total heterogeneity): 0 (SE = 0.0811)
## tau (square root of estimated tau^2 value):      0
## I^2 (total heterogeneity / total variability):   0.00%
## H^2 (total variability / sampling variability):  1.00
##
## Test for Heterogeneity:
## Q(df = 2) = 0.8440, p-val = 0.6557
##
## Model Results:
##
## estimate      se    zval    pval    ci.lb   ci.ub
##   0.0509  0.1633  0.3117  0.7553  -0.2692  0.3710
##
## ---
## Signif. codes:  0 '***' 0.001 '**' 0.01 '*' 0.05 '.' 0.1 ' ' 1
```

ROM 4 = Pronation

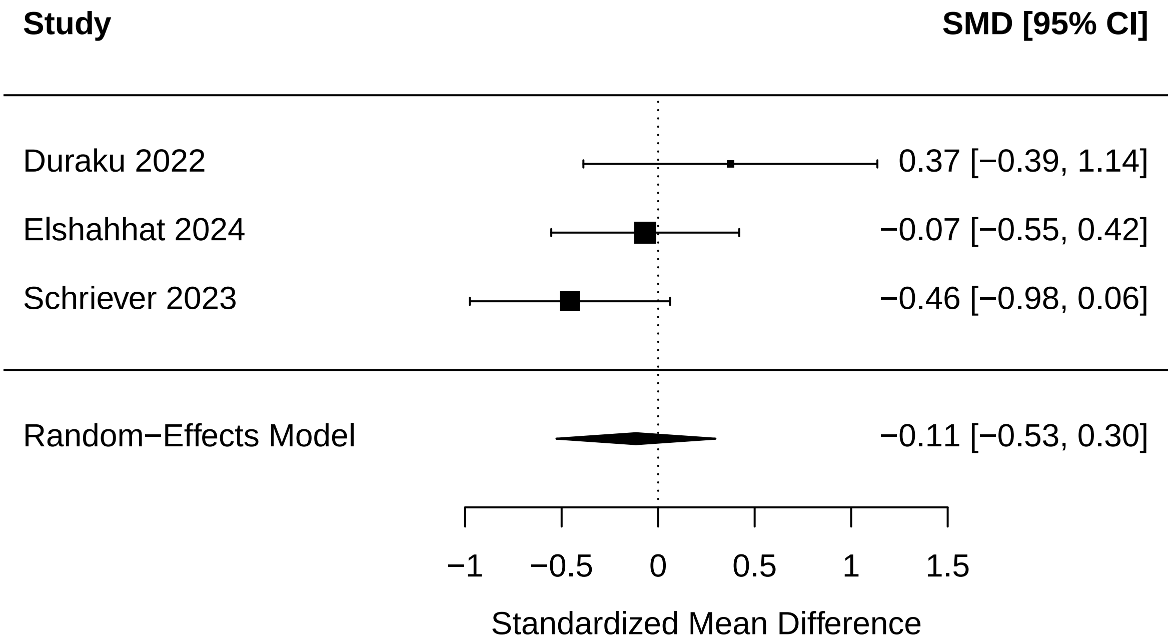


```
##
## Random-Effects Model (k = 3; tau^2 estimator: REML)
##
##   logLik  deviance       AIC       BIC      AICc
##  -0.9750    1.9500    5.9500    3.3362   17.9500
##
## tau^2 (estimated amount of total heterogeneity): 0.0479 (SE = 0.1338)
## tau (square root of estimated tau^2 value):      0.2189
## I^2 (total heterogeneity / total variability):   35.87%
## H^2 (total variability / sampling variability):  1.56
##
## Test for Heterogeneity:
## Q(df = 2) = 3.2740, p-val = 0.1946
##
## Model Results:
##
## estimate      se     zval    pval    ci.lb   ci.ub
##  -0.1149  0.2102  -0.5466  0.5847  -0.5269  0.2971
##
## ---
## Signif. codes:  0 '***' 0.001 '**' 0.01 '*' 0.05 '.' 0.1 ' ' 1
```

ROM 5 = Supination

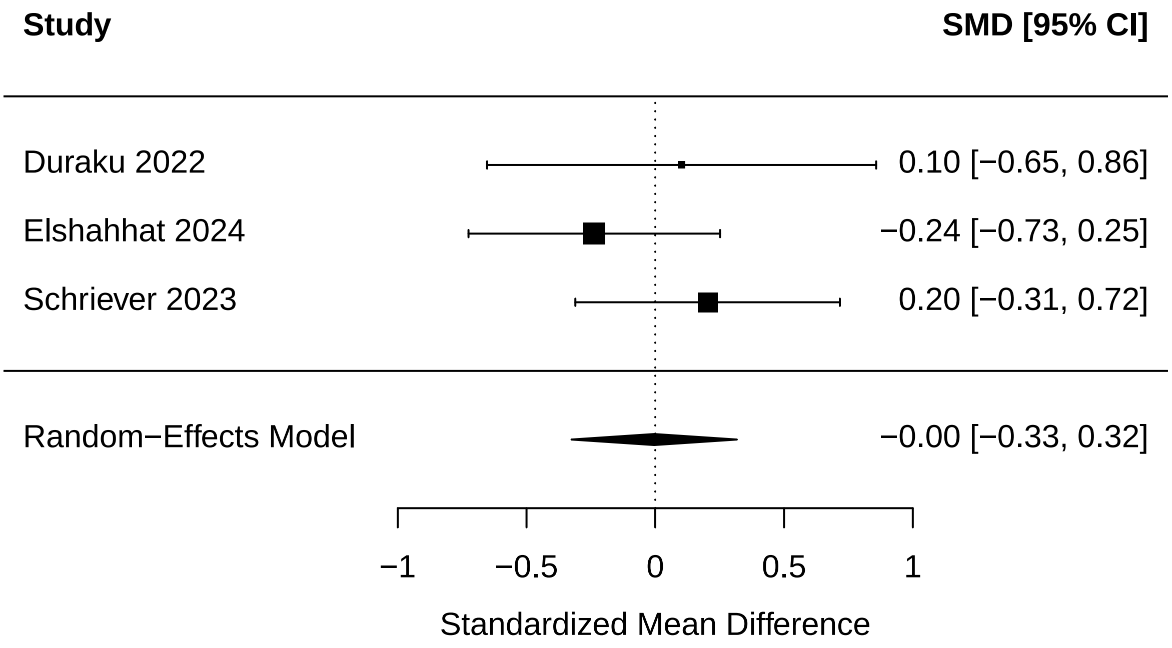


```
##
## Random-Effects Model (k = 3; tau^2 estimator: REML)
##
##   logLik  deviance       AIC       BIC      AICc
##  -0.2043    0.4087    4.4087    1.7950   16.4087
##
## tau^2 (estimated amount of total heterogeneity): 0.0002 (SE = 0.0815)
## tau (square root of estimated tau^2 value):      0.0147
## I^2 (total heterogeneity / total variability):   0.25%
## H^2 (total variability / sampling variability):  1.00
##
## Test for Heterogeneity:
## Q(df = 2) = 1.5746, p-val = 0.4551
##
## Model Results:
##
## estimate      se     zval    pval    ci.lb   ci.ub
##  -0.0043  0.1638  -0.0264  0.9789  -0.3253  0.3166
##
## ---
## Signif. codes:  0 '***' 0.001 '**' 0.01 '*' 0.05 '.' 0.1 ' ' 1
```

Arc of Motion Flexion-Extension

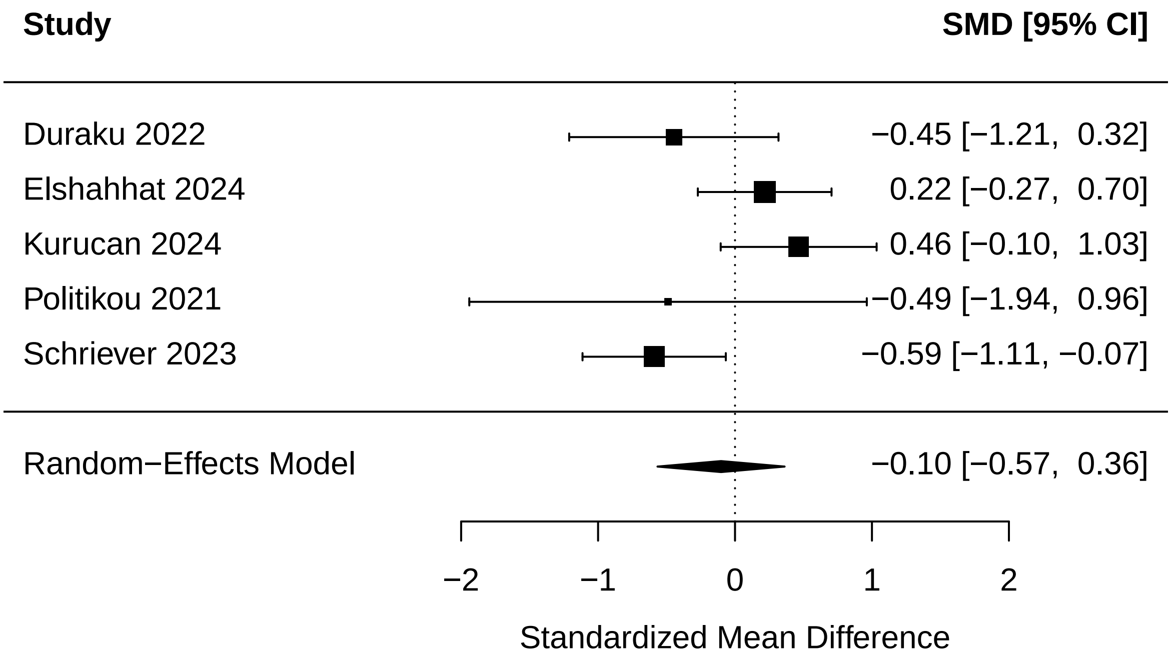


```
##
## Random-Effects Model (k = 5; tau^2 estimator: REML)
##
##   logLik  deviance       AIC       BIC      AICc
##  -3.0564    6.1129   10.1129    8.8855   22.1129
##
## tau^2 (estimated amount of total heterogeneity): 0.1559 (SE = 0.1939)
## tau (square root of estimated tau^2 value):      0.3949
## I^2 (total heterogeneity / total variability):   59.47%
## H^2 (total variability / sampling variability):  2.47
##
## Test for Heterogeneity:
## Q(df = 4) = 9.7633, p-val = 0.0446
##
## Model Results:
##
## estimate      se     zval    pval    ci.lb   ci.ub
##  -0.1019  0.2369  -0.4303  0.6670  -0.5663  0.3624
##
## ---
## Signif. codes:  0 '***' 0.001 '**' 0.01 '*' 0.05 '.' 0.1 ' ' 1
```

Arc of Motion Radial-Ulnar

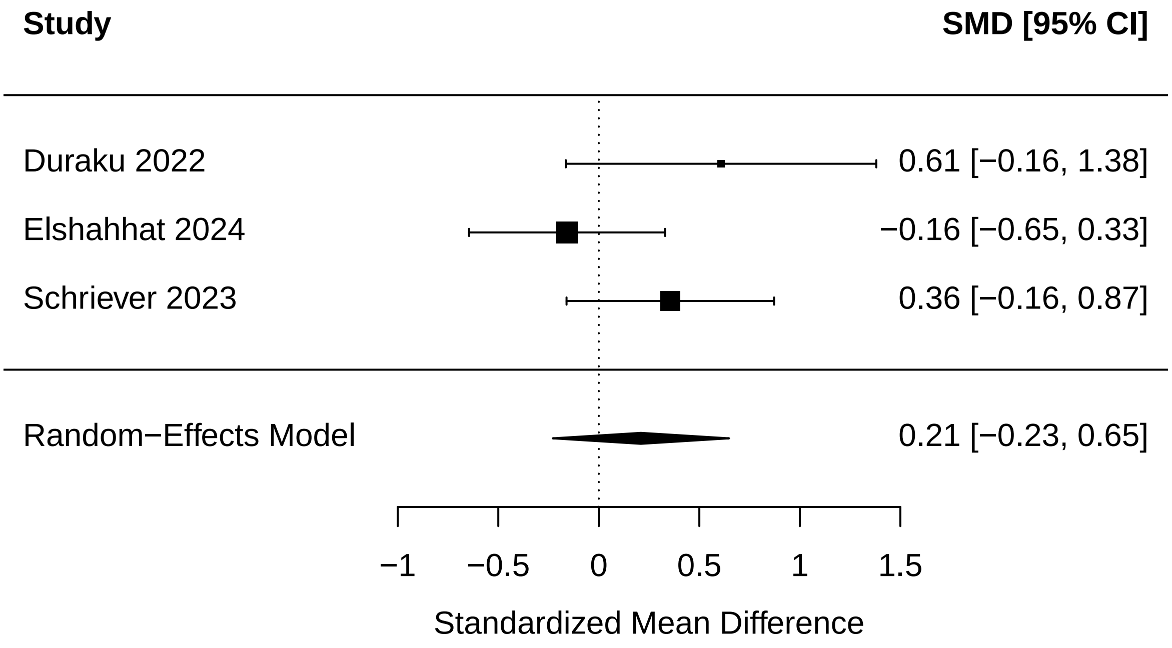


```
##
## Random-Effects Model (k = 3; tau^2 estimator: REML)
##
##   logLik  deviance       AIC       BIC      AICc
##  -0.9631    1.9263    5.9263    3.3125   17.9263
##
## tau^2 (estimated amount of total heterogeneity): 0.0643 (SE = 0.1516)
## tau (square root of estimated tau^2 value):      0.2536
## I^2 (total heterogeneity / total variability):   42.77%
## H^2 (total variability / sampling variability):  1.75
##
## Test for Heterogeneity:
## Q(df = 2) = 3.4718, p-val = 0.1762
##
## Model Results:
##
## estimate      se    zval    pval    ci.lb   ci.ub
##   0.2093  0.2238  0.9351  0.3497  -0.2294  0.6480
##
## ---
## Signif. codes:  0 '***' 0.001 '**' 0.01 '*' 0.05 '.' 0.1 ' ' 1
```

Arc of Motion Pronation-Supination

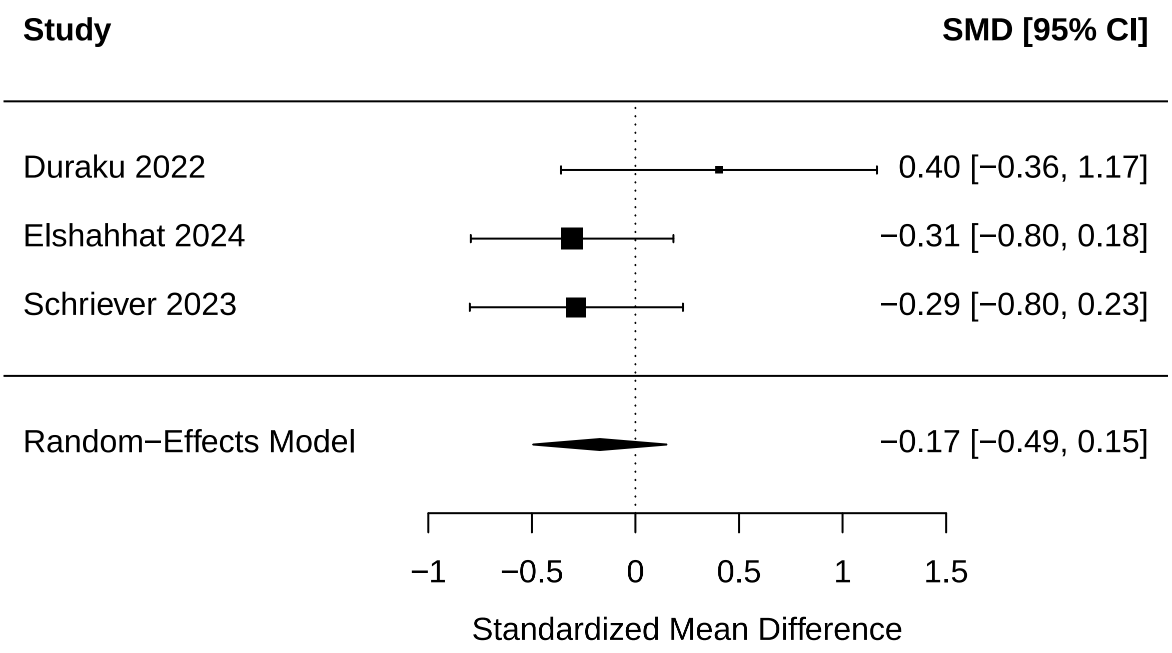


```
##
## Random-Effects Model (k = 3; tau^2 estimator: REML)
##
##   logLik  deviance       AIC       BIC      AICc
##  -0.7578    1.5156    5.5156    2.9019   17.5156
##
## tau^2 (estimated amount of total heterogeneity): 0.0000 (SE = 0.0819)
## tau (square root of estimated tau^2 value):      0.0023
## I^2 (total heterogeneity / total variability):   0.01%
## H^2 (total variability / sampling variability):  1.00
##
## Test for Heterogeneity:
## Q(df = 2) = 2.6603, p-val = 0.2644
##
## Model Results:
##
## estimate      se     zval    pval    ci.lb   ci.ub
##  -0.1718  0.1641  -1.0469  0.2951  -0.4935  0.1498
##
## ---
## Signif. codes:  0 '***' 0.001 '**' 0.01 '*' 0.05 '.' 0.1 ' ' 1
```
