# Supplementary material for: Capitolunate arthrodesis versus four-corner fusion for advanced wrist collapse: a systematic review and meta-analysis
Source: J Orthop Surg Res. 2026 Mar 13;21:268. doi: 10.1186/s13018-026-06747-x (PMC13097632; doi:10.1186/s13018-026-06747-x)
Supplement: Supplementary file 2 — Supplementary Material 2. Full Search Strategies This file contains the complete search strategies used in PubMed, EMBASE, and Web of Science, including all keywords, MeSH terms, Boolean operators, and filters applied in the systematic search conducted on December 27, 2024. [file 13018_2026_6747_MOESM2_ESM.pdf]

Search Strategy

Systematic Review and Meta-Analysis

Clinical Outcomes of Capitolunate Arthrodesis: A Systematic Review and Meta-Analysis

Search Strategy

	**Population**		**Intervention**	**Outcome**
**Our Study**	SLAC/SNAC Wrist<br>(Adult 18+)		Capitolunate arthrodesis	Pain Score, Functional Improvement, Range of Motion Preserved, Operative Time, Complication Rates
**Keywords**	Adults	“Scaphoid nonunion advanced collapse”<br>“Scapholunate advanced collapse”<br>“Scaphoid Bone”<br>“Osteoarthritis*”	“CLA”<br>“Capitolunate arthrodesis”<br>“Capitolunate Arthrodeses”<br>“Lunocapitate Arthrodesis”<br>“Lunocapitate Arthrodeses”<br>“fusion*”	“Pain-free”<br>“Pain free”<br>“Pain score”<br>“Complicat*”<br>“Musculoskeletal Pains”<br>“Musculoskeletal Pain”

**MeSH**	"Humans"[Mesh]<br>"Adult"[Mesh]	"Wrist"[Mesh]<br>"Wrist Joint"[Mesh]<br>"Arthritis"[Mesh]<br>"Osteoarthritis"[Mesh]	"Arthrodesis"[Mesh]	"Musculoskeletal Pain"[Mesh]<br>"Pain, Postoperative"[Mesh]<br>"Arthralgia"[Mesh]<br>"Range of Motion, Articular"[Mesh]<br>"Treatment Outcome"[Mesh]

Databases

PubMed, EMBASE, Web of Science

Date of 1st Search

Dec. 27th, 2024
